# Transcranial Direct-Current Stimulation Regulates MCT1-PPA-PTEN-LONP1 Signaling to Confer Neuroprotection After Rat Cerebral Ischemia–Reperfusion Injury

**DOI:** 10.1007/s12035-022-03051-7

**Published:** 2022-10-03

**Authors:** Xiangyi Kong, Wenjie Hu, Yu Cui, Jingchen Gao, Xujin Yao, Jinyang Ren, Tao Lin, Jiangdong Sun, Yunyi Gao, Xiaohua Li, Hui Wang, Huanting Li, Fengyuan Che, Qi Wan

**Affiliations:** 1 Institute of Neuroregeneration & Neurorehabilitation, Department of Neurosurgery, Qingdao University, 308 Ningxia Street, Qingdao, 266071 China; 2 Department of Biological Science, Jining Medical University, Rizhao, Shandong China; 3 Central Laboratory, Department of Neurology, Linyi People’s Hospital, Qingdao University, 27 East Jiefang Road, Linyi, Shandong China; 4 Qingdao High-tech Industrial Development District, Qingdao Gui-Hong Intelligent Medical Technology Co. Ltd, 7 Fenglong Road, Qingdao, China

**Keywords:** Propionic acid, Cerebral ischemic stroke, Neuroprotection, LONP1, tDCS

## Abstract

Propionic acid (PPA) is a critical metabolite involved in microbial fermentation, which functions to reduce fat production, inhibit inflammation, and reduce serum cholesterol levels. The role of PPA in the context of cerebral ischemia–reperfusion (I/R) injury has yet to be clarified. Increasing evidence indicate that transcranial direct-current stimulation (tDCS) is a safe approach that confers neuroprotection in cerebral ischemia injury. Here, we show that the levels of PPA were reduced in the ischemic brain following a rat cerebral I/R injury and in the cultured rat cortical neurons after oxygen–glucose deprivation (OGD), an in vitro model of ischemic injury. We found that the decreased levels of transporter protein monocarboxylate transporter-1 (MCT1) were responsible for the OGD-induced reduction of PPA. Supplementing PPA reduced ischemia-induced neuronal death after I/R. Moreover, our results revealed that the neuroprotective effect of PPA is mediated through downregulation of phosphatase PTEN and subsequent upregulation of Lon protease 1 (LONP1). We demonstrated that direct-current stimulation (DCS) increased MCT1 expression and PPA level in OGD-insulted neurons, while tDCS decreased the brain infarct volume in the MCAO rats via increasing the levels of MCT1 expression and PPA. This study supports a potential application of tDCS in ischemic stroke.

**Supplementary Information:**

The online version contains supplementary material available at 10.1007/s12035-022-03051-7.

## Introduction 

Short-chain fatty acids (SCFA) are the main metabolites for anaerobic fermentation in the mammal intestinal microbiome. These fatty acids act as gene expression regulators and signal molecules that are recognized by specific receptors. As energy sources, they are effectively absorbed by intestinal mucosa and play an important role in human physiological functions [1]. Relevant SCFAs include acetic, propionic, butyric, valeric, succinic, and caproic acid [2]. The three main short-chain fatty acids, acetic acid, propionic acid, (PPA) and butyric acid, have significantly varied effects on human physiology. Butyric acid is metabolized as a source of source, propionic acid contributes to gluconeogenesis in the liver, and acetate exits in the highest concentration in the blood [3]. However, the mechanism of PPA in the brain is still poorly studied. Recent studies have shown that PPA can regulate the conduction process of various neuronal cell signals once it enters the brain through the blood–brain barrier, including energy and lipid metabolism, as well as the synthesis and release of neurotransmitters [3]. PPA has been shown to inhibit the expression of AKT and PTEN after crossing the blood–brain barrier, thus promoting cell proliferation [4]. The impact of PPA in the context of ischemic stroke, however, has yet to be explored.

To explore the main pathway of PPA in and out of cells, we studied monocarboxylate transporter 1 (MCT1), a channel protein. MCT1 is a type of MCTs; its main function is responsible for the up-taking and release of energy substrates including acetate, propionate, and pyruvate [5]. MCT1 is expressed in most human cells. The primary ketone body, β-hydroxybutyrate, is also a substrate for MCT1; during starvation, the circulating levels of this metabolite markedly increase to millimolar concentrations from hepatic synthesis to serve as the primary metabolic fuel for neurons in place of glucose. Moreover, this metabolite crosses the blood–brain barrier via MCT1. Increased levels of MCT1 have been shown to reduce ischemia–reperfusion injury, but the exact mechanism remains unclear.

Lonp1 is an important stress response protease that is predominantly concentrated in the mitochondrial matrix. LonP1 acts as a major regulator of mitochondrial metabolism, mediating mitochondrial dynamic homeostasis, metabolism, and bioenergetics [6]. Furthermore, it has a critical role in the maintenance and repair of mitochondrial DNA [7–9]. Recent studies have found that deletion of the LONP1 gene, encoding LONP1 protease, can cause a variety of human diseases, including mitochondrial related diseases, which can result in significant neurological impairment and multiple organ dysfunction [10]. LonP1 has also been shown to be involved in the pathogenesis of cancer where it promotes the growth of primary and metastatic tumors by regulating cell death [11–13], ROS levels, and metabolic programming [8, 14]. Studies have shown that Akt phosphorylation enhances the protease activity of mitochondrial LONP1 under hypoxic conditions [15]. The role of LONP1 in the context of ischemic stroke, however, remains to be elucidated.

Transcranial direct-current stimulation (tDCS) is a noninvasive brain stimulation technique that can be used to modulate central nervous system excitability in humans to promote post-stroke recovery [16–18]. We have previously demonstrated that tDCS treatment promotes axonal regeneration and neuronal growth and that physiological intensity of DC can guide nerve stem/progenitor cell (NSPC) migration [16]. In rats with ischemic injury, cathodal electrodes placed on the ischemic side of the brain with low-intensity direct-current stimulation increase the number of endogenous neural stem cells in the subependymal ventricular zone (SVZ) and promotes NSPC migration towards the ischemic area, thereby reducing ischemia–reperfusion injury [19]. These studies raise the potential for tDCS as a noninvasive treatment for neurological diseases. However, the mechanism underlying tDCS-induced neuroprotection remains unclear.

Here, we employed a rat middle cerebral artery occlusion (MCAO) model system to explore the functional role of PPA within damaged neurons following cerebral ischemia–reperfusion (I/R) injury. We found that following cerebrovascular injury, intracortical PPA was reduced. Under oxygen–glucose deprivation (OGD) conditions, we observed a consequential decrease in intracortical neuron PPA levels. The exogenous addition of PPA resulted in the alleviation of neuronal injury. Mechanistically, PPA was found to act upstream of phosphatase PTEN, with the LONP1 functioning as a downstream phosphatase PTEN target that has a PPA-induced neuroprotective role within the brain following I/R injury. Notably, DCS treatment resulted in increased PPA levels within cortical cells in OGD model rats. tDCS treatment further decreased the cerebral infarct volume in rats following cerebral I/R injury via the increase of intraneuronal PPA concentrations.

## Results

### PPA Levels Are Decreased Following Rat Cerebral I/R Injury

**Fig. 1:**
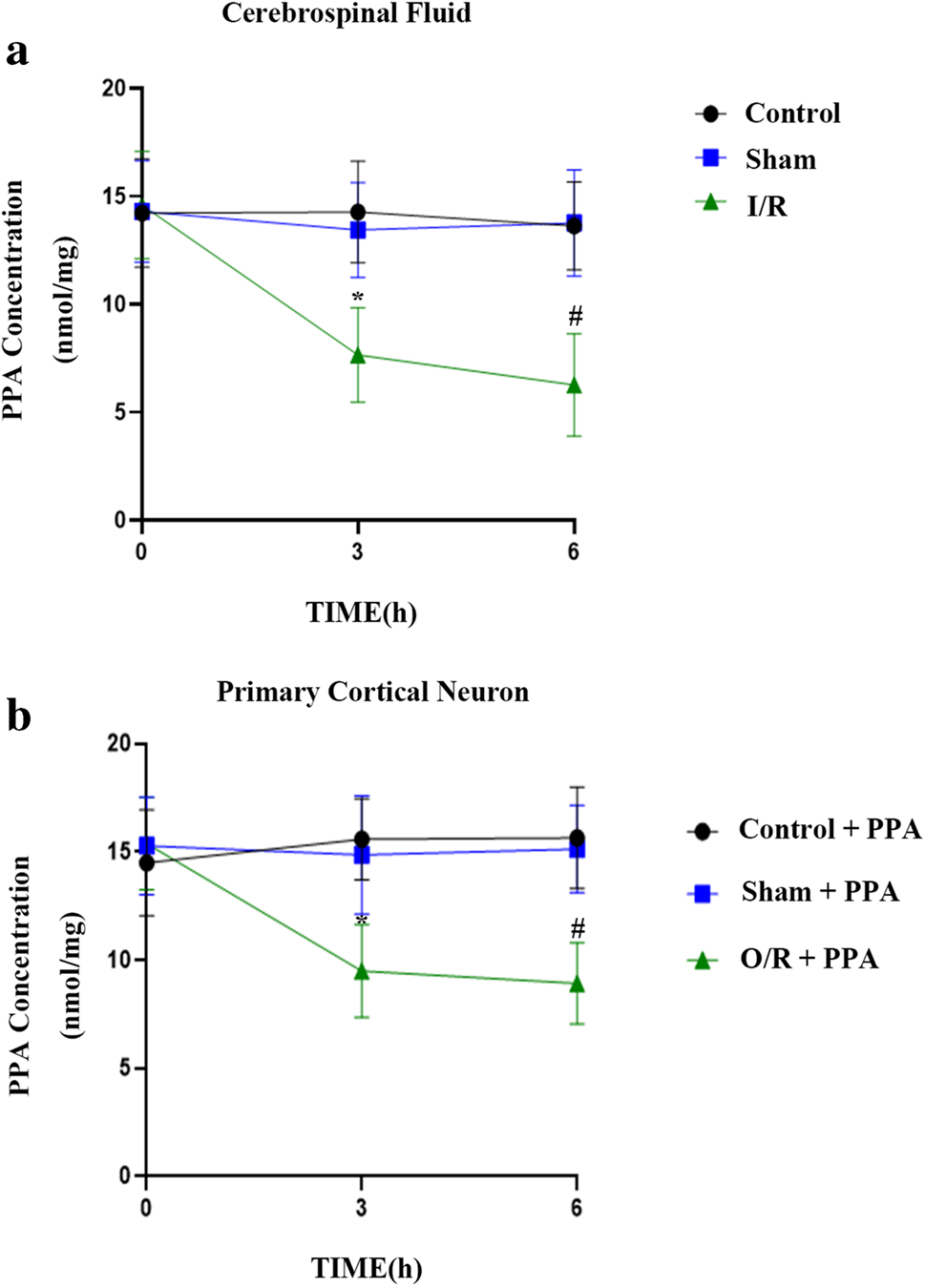
Intraneuronal PPA is decreased after rat cerebral ischemia–reperfusion injury. **a** HPLC analyses of PPA levels within rat cerebrospinal fluid following cerebral ischemia modeling revealed reduced levels of extra-neuronal PPA after cerebral ischemia–reperfusion injury. Levels of PPA were measured at 0, 3, and 6 h following a 1-h occlusion period (*n* = 6/ group, ^*^p < 0.05 vs. 3 h control, ^#^*p* < 0.05 vs. 6 h control, one-way ANOVA). **b** HPLC analyses of PPA levels in primary cultured neurons following OGD injury revealed decreased intraneuronal PPA levels following I/R injury. Primary cortical neurons were collected for analysis at 0, 3, and 6 h following reperfusion. (*n* = 6/group, ^*^p < 0.05 vs. 3 h control, ^#^*p* < 0.05 vs. 6 h control, one-way ANOVA)

To explore the functional importance of PPA in the context of cerebral I/R injury, we began by assessing levels of PPA in CSF samples collected from rats at 0, 3, or 6 h following MCAO modeling via HPLC. This analysis revealed significantly decreased PPA levels within the CSF following cerebral I/R injury (Fig. 1a), suggesting that the extracellular levels of this PPA reduced in damaged brain tissue following ischemic injury.

To extend the above results to an in vitro system, we assessed PPA levels within cultured rat neurons following OGD treatment via HPLC. Neurons cannot produce PPA; thus, to verify the effect of PPA on neurons, we added PPA to neurons before OGD. Consistently, intraneuronal PPA levels were decreased following OGD-induced injury (Fig. 1b).

## OGD-Induced Decrease of Membrane MCT1 Leads to the Reduction of Intraneuronal PPA

To better clarify the mechanisms whereby OGD induced intraneuronal PPA decrease, we next assessed the functional importance of MCT1 as a regulator of PPA concentrations within neurons. HPLC analyses revealed that the treatment of cells with the MCT1 inhibitor, AZD3965, markedly suppressed intraneuronal PPA levels (Supplementary Fig. 1a). Western blotting further revealed that the levels of MCT1, associated with neuronal membranes, decrease following I/R modeling (Supplementary Fig. 1b). This suggested decreased membrane-associated MCT1 mediated the accumulation of PPA within neurons following OGD injury.

## Supplementing PPA Is Neuroprotective in Cerebral I/R Injury

**Fig. 2:**
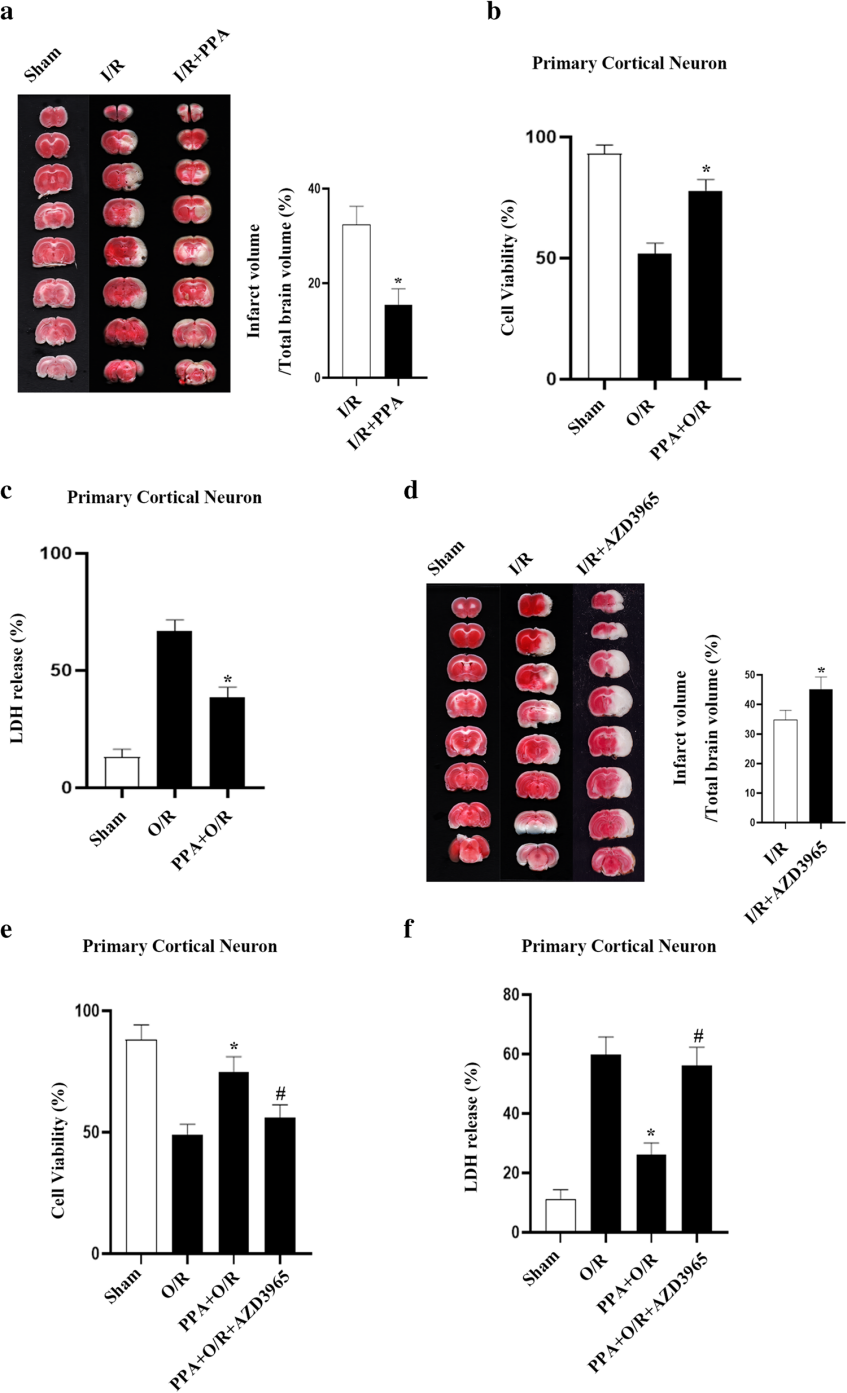
Increased intraneuronal PPA reduced neuronal injury following cerebral ischemia–reperfusion in rats. **a** TTC staining revealed that PPA supplementation resulted in a decrease in infarct volume. PPA was injected intraperitoneally 1 h after reperfusion (*n* = 6/group, ^*^p < 0.05 vs. I/R, one-way ANOVA). **b** Cellular viability assays revealed that PPA treatment decreased primary cultured neuron death following OGD injury. PPA (1 mM) was administered to primary neuronal cultures following reoxygenation for 1 h (*n* = 6/group, F (2, 15) = 22.4, ^*^p < 0.05 vs. O/R, one-way ANOVA). **c** LDH release assays revealed that PPA decreased primary cultured neuron death following OGD injury. Primary neuronal cultures were treated with PPA (50 μM) following 1 h of reoxygenation (*n* = 6/group, F (2, 15) = 42.2, ^*^p < 0.05 vs. O/R, one-way ANOVA). **d** TTC staining of brain sections revealed that intravenous injection of the MCT1 inhibitor AZD3965 (10 μM, 1 μL) prior to MCAO modeling increased the infarct area (*n* = 6 in each group, ^*^p < 0.05 vs. I/R, one-way ANOVA). **e** Cellular activity assays using primary cultured neurons revealed that MCT1 inhibition increased OGD-induced neuronal death. Neurons were treated for 1 h with AZD3965 (10 μM) prior to OGD modeling (*n* = 6/group, F (3, 20) = 10.45, ^*^p < 0.05 vs. O/R, ^#^*p* < 0.05 vs. PPA + O/R, one-way ANOVA). **f** LDH release assays using primary cultured neurons revealed that MCT1 inhibition increased OGD-induced neuronal death. Neurons were treated for 1 h with AZD3965 (10 μM) prior to OGD modeling (*n* = 6/group, F (3, 20) = 22.31, ^*^p < 0.05 vs. O/R, ^#^*p* < 0.05 vs. PPA + O/R, one-way ANOVA)

To determine the role of PPA on neuronal survival in the context of cerebral I/R injury, the effect of PPA treatment in the rat MCAO model was assessed. Intraperitoneal injection of PPA decreased the average infarct volume in the stroke animals (Fig. 2a). Similarly, PPPA treatment reduced the neuronal death in cortical cultures following OGD injury (Fig. 2b–c). We next assessed the effect of AZD3965 treatment in the rat MCAO model. We found that AZD3965 increased the average infarct volume after I/R injury (Fig. 2d) and reduced the neuronal viability in cortical cultures following OGD injury (Fig. 2e–f). Together, these data indicate that PPA is neuroprotective in cerebral I/R injury. PPA supplement is a potential therapeutic approach for acute ischemic stroke.

## The Neuroprotective Effect of PPA Is Mediated through Downregulation of PTEN and Upregulation of LONP1

**Fig. 3:**
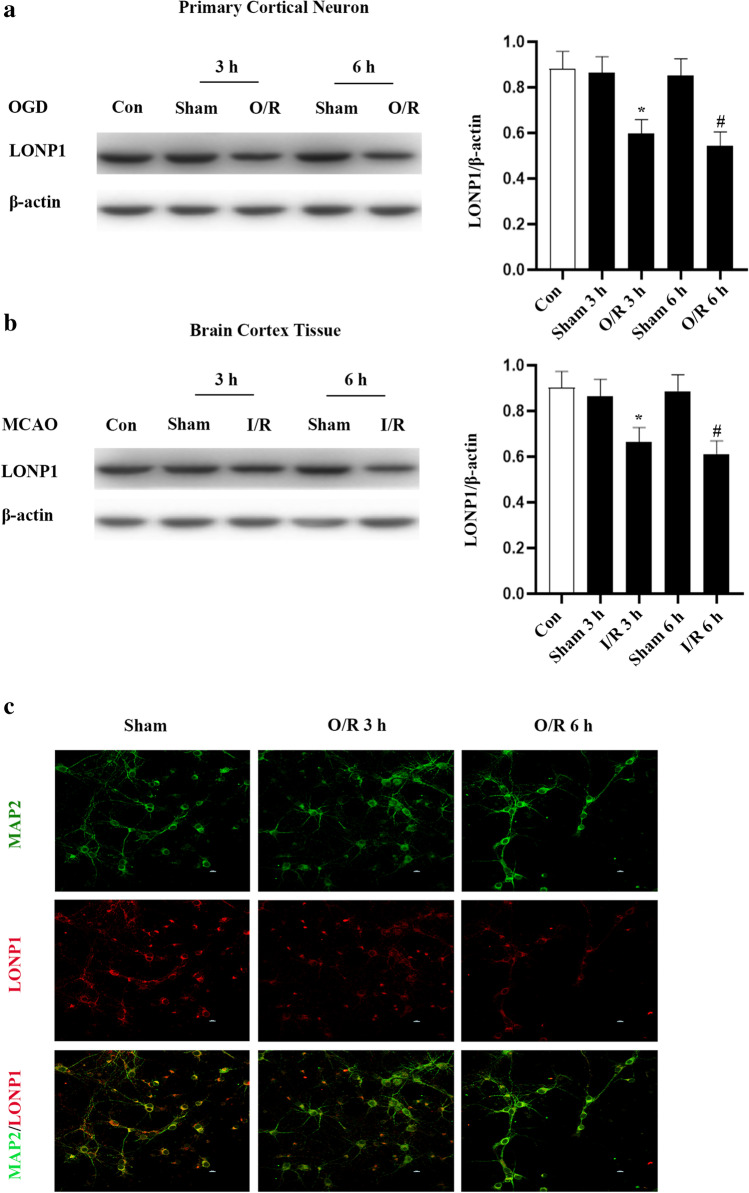
LONP1 levels are decreased in damaged neurons following ischemia-repression injury. **a** Western blotting conducted at 3 and 6 h following OGD injury revealed reduced LONP1 levels in primary cortical neurons following OGD injury. Primary cortical neurons were collected for analysis at 3 and 6 h following OGD for 1 h (*n* = 6/group, F (4, 25) = 5.56, ^*^p < 0.05 vs. sham 3 h, ^#^*p* < 0.05 vs. sham 6 h, one-way ANOVA). **b** Western blotting revealed reduced LONP1 levels within the rat cerebral cortex following cerebral ischemia–reperfusion injury. Peri-infarct cortical tissue was collected for analysis at 3 and 6 h following occlusion for 1 h (*n* = 6/group, F (4, 25) = 3.99, ^*^p < 0.05 vs. sham 3 h, ^#^*p* < 0.05 vs. sham 6 h, one-way ANOVA). **c** Immunofluorescent LONP1 staining suggested a reduction in LONP1 levels within neurons following OGD injury. Staining was performed at 3 and 6 h following a 1-h OGD injury period. LONP1 (red) was primarily detected within the cytoplasm, and neurons were labeled with MAP2 (green) (*n* = 6/group)

To elucidate the mechanisms whereby PPA reduces neuronal death, in the context of I/R injury, we next assessed LONP1 expression in our experimental systems. LONP1 was detectable in rat brain samples and was downregulated in tissues from the cortical penumbral region following MCAO modeling and in primary neuronal cultures following OGD treatment (Supplementary Fig. 2a; Fig. 3a–c). We found that the LONP1 inhibitor, bortezomib, promoted the death of OGD-induced cortical neurons (Supplementary Fig. 3a–b).

**Fig. 4:**
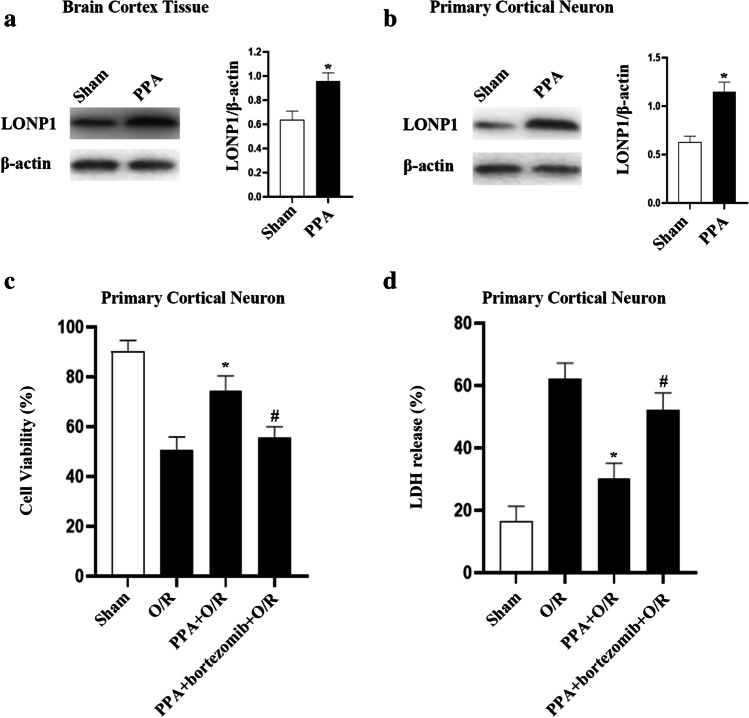
PPA upregulates LONP1 and reduces neuronal death following OGD injury. **a** Western blotting conducted following the intraperitoneal injection of PPA (0.2 mL/10 g) 6 h prior to cortical extraction revealed that PPA induced LONP1 upregulation within the rat cerebral cortex. (*n* = 6/group, ^*^p < 0.05 vs. sham by Student’s *t*-test). **b** Western blotting conducted following the 6-h treatment of primary cultured neurons with PPA (1 mM) revealed that PPA induced LONP1 upregulation within these cells (*n* = 6/group, ^*^p < 0.05 vs. sham by Student’s *t*-test). **c** Cell survival experiments revealed that the inhibition of LONP1 resulted in more pronounced PPA-induced neuronal damage following OGD injury. Primary cultured neurons were treated with the LONP1 inhibitor bortezomib for 72 h prior to OGD injury and were treated with PPA following a 1-h reoxygenation period (*n* = 6/group, F (3, 20) = 12.78, ^*^p < 0.05 vs. O/R, ^#^*p* < 0.05 vs. PPA + O/R, one-way ANOVA). **d** LDH release assays revealed that the inhibition of LONP1 resulted in more pronounced PPA-induced neuronal damage following OGD injury. Primary cultured neurons were treated with the LONP1 inhibitor bortezomib for 72 h prior to OGD injury and were treated with PPA following a 1-h reoxygenation period (*n* = 6/group, F (3, 20) = 16.41, ^*^p < 0.05 vs. O/R, ^#^*p* < 0.05 vs. PPA + O/R, one-way ANOVA)

Given that PPA can regulate PI3K/Akt signaling and Akt phosphorylation enhances the protease activity of mitochondrial LonP1, under hypoxia conditions, we hypothesized that PPA may function as an upstream LONP1 signaling regulator [15]. We found that the intraperitoneal administration of PPA in rats increased cortical LONP1 levels (Fig. 4a). In line with these results, treatment with PPA increased LONP1 expression in cultured neurons (Fig. 4b). As such, PPA functions as an upstream regulator to promote LONP1 upregulation.

Given that PPA induced LONP1 upregulation, we next explored the ability of PPA to reduce OGD-induced neuronal death through a mechanism dependent on LONP1 upregulation. We found that LONP1 inhibition exacerbated such neuronal injury following OGD (Fig. 4c–d). These results suggest that LONP1 mediates PPA-induced neuronal protection.

## Downregulating PTEN Promotes LONP1 Upregulation to Mediate PPA-Induced Neuroprotection

**Fig. 5:**
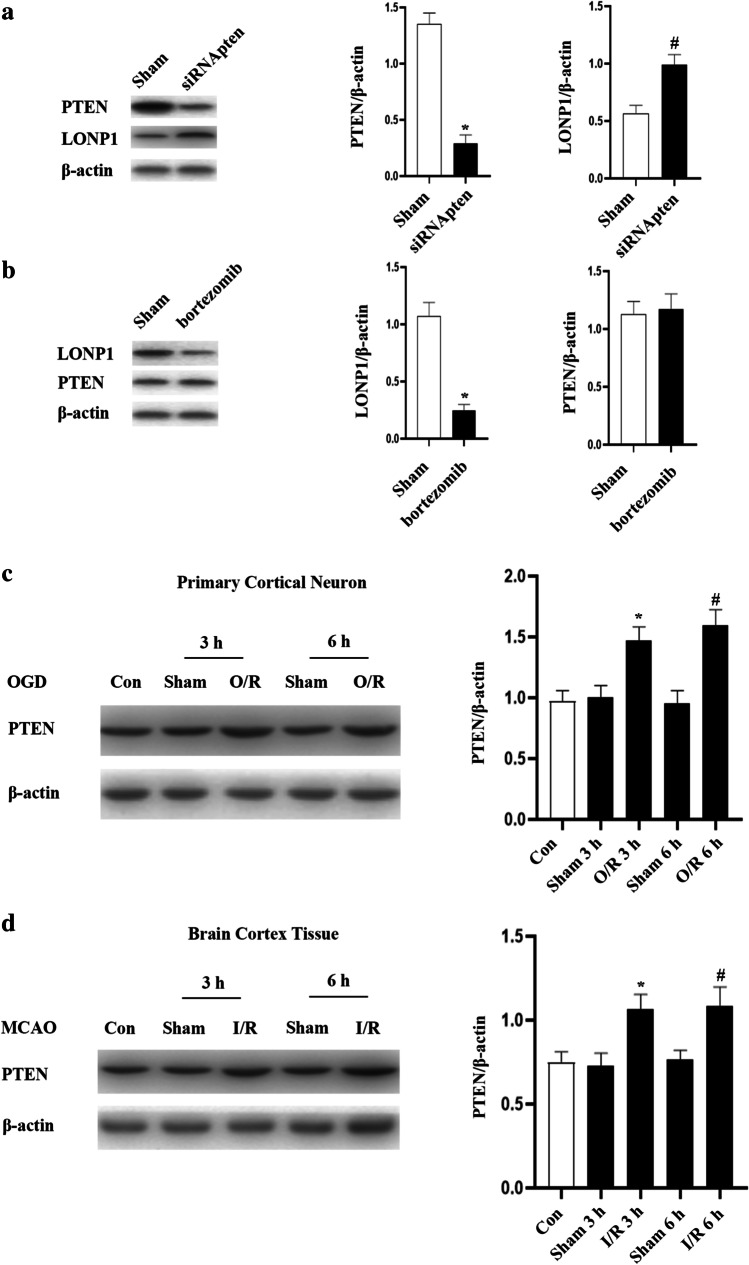
The downregulation of PTEN enhances LONP1 expression following OGD injury. **a** Western blotting analyses indicated that PTEN regulates the expression of LONP1 within neurons. Primary neurons were treated with siRNA of PTEN for 72 h, after which Western blotting was conducted (*n* = 6/group, ^*^p < 0.05 vs. sham, ^#^*p* < 0.05 vs. sham, Student’s *t*-test). **b** Western blotting indicated that LONP1 had no impact on PTEN expression within neurons. Neurons were treated with bortezomib for 72 h, after which Western blotting was conducted (*n* = 6/group, ^*^p < 0.05 vs. sham, Student’s *t*-test). **c** Western blotting conducted at 3 and 6 h following reoxygenation indicated that neurons exhibited PTEN upregulation in cultured cortical neurons (*n* = 6/group, F (4, 25) = 8.37, ^*^p < 0.05 vs. sham 3 h, ^#^*p* < 0.05 vs. sham 6 h, one-way ANOVA). **d** Western blotting revealed increased PTEN levels within the rat cerebral cortex following cerebral ischemia–reperfusion injury. Peri-infarct cortical tissue was collected for analysis at 3 and 6 h following occlusion for 1 h (*n* = 6/group, F (4, 25) = 4.72, ^*^p < 0.05 vs. sham 3 h, ^#^*p* < 0.05 vs. sham 6 h, one-way ANOVA)

PTEN is an essential regulator of cellular viability [20–22]. PTEN can lead to cardiac injury in diabetic patients by acting on the PI3K/AKT pathway. Akt phosphorylation enhances the protease activity of mitochondrial LonP1 under hypoxia conditions [15, 23]. We hypothesized that PTEN functions as a mediator of neuronal damage in a LONP1-dependent manner, following OGD. Our data showed that LONP1 levels rose following PTEN siRNA (Fig. 5a). In contrast, LONP1 inhibition had no impact on PTEN levels (Fig. 5b). Thus, LONP1 is a downstream target of PTEN.

**Fig. 6:**
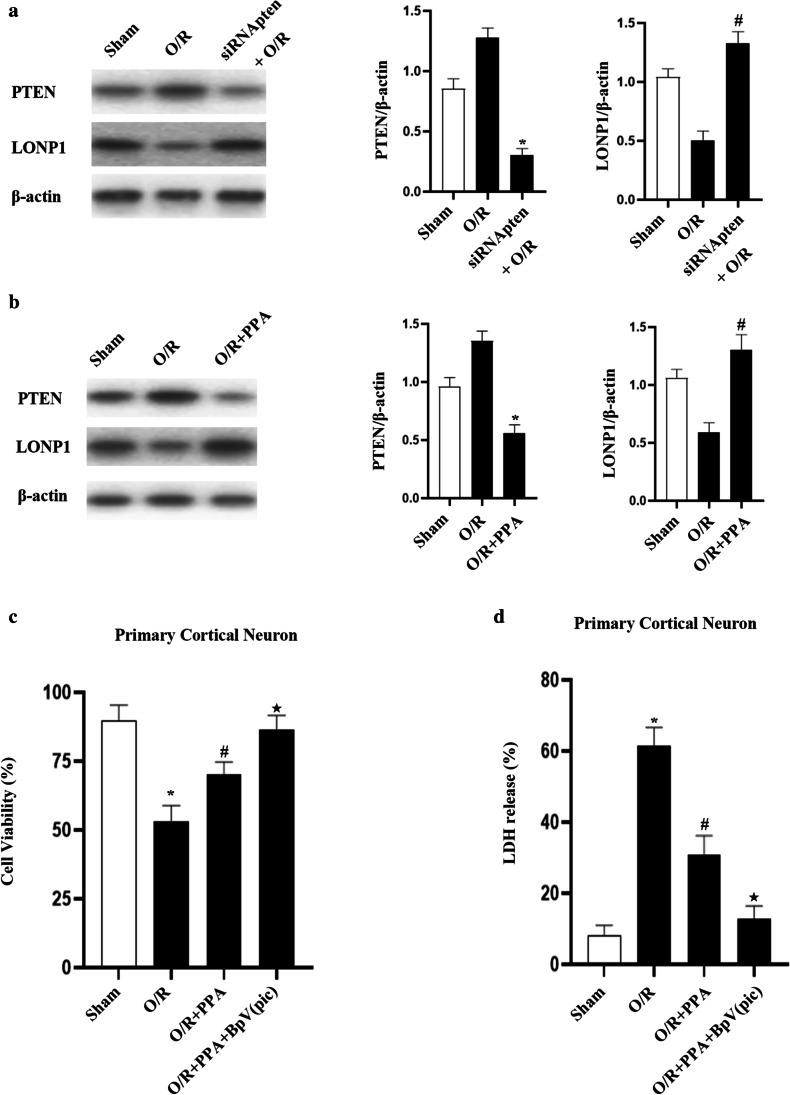
PPA promotes PTEN downregulation and LONP1 upregulation within neurons following OGD injury. **a** Western blotting indicated that PTEN controls LONP1 expression within neurons following OGD injury. Neurons were treated siRNA of PTEN 72 h prior to a 1-h hypoxic treatment period and were then harvested for Western blotting following a 6-h reoxygenation period (*n* = 6/group, ^*^p < 0.05 vs. O/R, ^#^*p* < 0.05 vs. O/R, one-way ANOVA). **b** Western blotting revealed that PPA decreased PTEN expression and increased LONP1 expression following OGD injury. After a 1-h reoxygenation period, primary neuron cultured were treated with PPA (1 mM), with Western blotting being conducted at 6 h post-reoxygenation (*n* = 6/group, ^*^p < 0.05 vs. O/R, ^#^*p* < 0.05 vs. O/R, one-way ANOVA). **c** Cell survival assays revealed that PTEN-mediated PPA promoted neuronal death following OGD injury, with cells having been treated with the PTEN inhibitor BpV(pic) 1 h prior to OGD treatment (*n* = 6/group, F (3, 20) = 10.24, ^*^p < 0.05 vs. sham, ^#^*p* < 0.05 vs. O/R, ^★^*p* < 0.05 vs. PPA + O/R, one-way ANOVA). **d** LDH release assays revealed that PTEN-mediated PPA promoted neuronal death following OGD injury, with cells having been treated with the PTEN inhibitor BpV(pic) 1 h prior to OGD treatment (**n** = 6/group, F (3, 20) = 31.6, ^*^p < 0.05 vs. sham, ^#^**p** < 0.05 vs. O/R, ^★^**p** < 0.05 vs. PPA + O/R, one-way ANOVA)

We further showed PTEN upregulation following ischemia–reperfusion injury (Fig. 5c–d). The siRNA of PTEN resulted in LONP1 upregulation in these cells (Fig. 6a). These data further support a model wherein PTEN upregulation results in LONP1 downregulation following OGD injury.

To clarify whether LONP1 functions as a downstream target of PTEN when facilitating OGD-induced PPA-mediated neuronal death, we next demonstrated that PPA treatment resulted in PTEN downregulation and consequential LONP1 upregulation (Fig. 6b). Cell viability and LDH release assays revealed that the treatment of cells with the PTEN inhibitor BpV (pic) resulted in PPA having a protective effect on neuronal injury as well as it stopping cellular death (Fig. 6c–d). Together, these results suggest that the PTEN-LONP1 signaling pathway can control OGD-induced neuronal death through mechanisms tied to increased PPA levels.

## DCS Increased Membrane MCT1 Expression and Neuronal PPA Levels After OGD

MCT1 is expressed not only in the apical membrane but also in the basolateral membrane, suggesting a role in the efflux of SCFA from the cells to the serosal side. The electroneutral co-transport of SCFA^−^ with H^+^ via MCT1 could occur in either direction (influx or efflux) depending on the transmembrane concentration gradient for a given SCFA^−^ [24]. These data suggest that an increase of MCT1 reduces ischemic damage following I/R injury in the rat brain through the PPA-PTEN-LONP1 signaling axis.

**Fig. 7:**
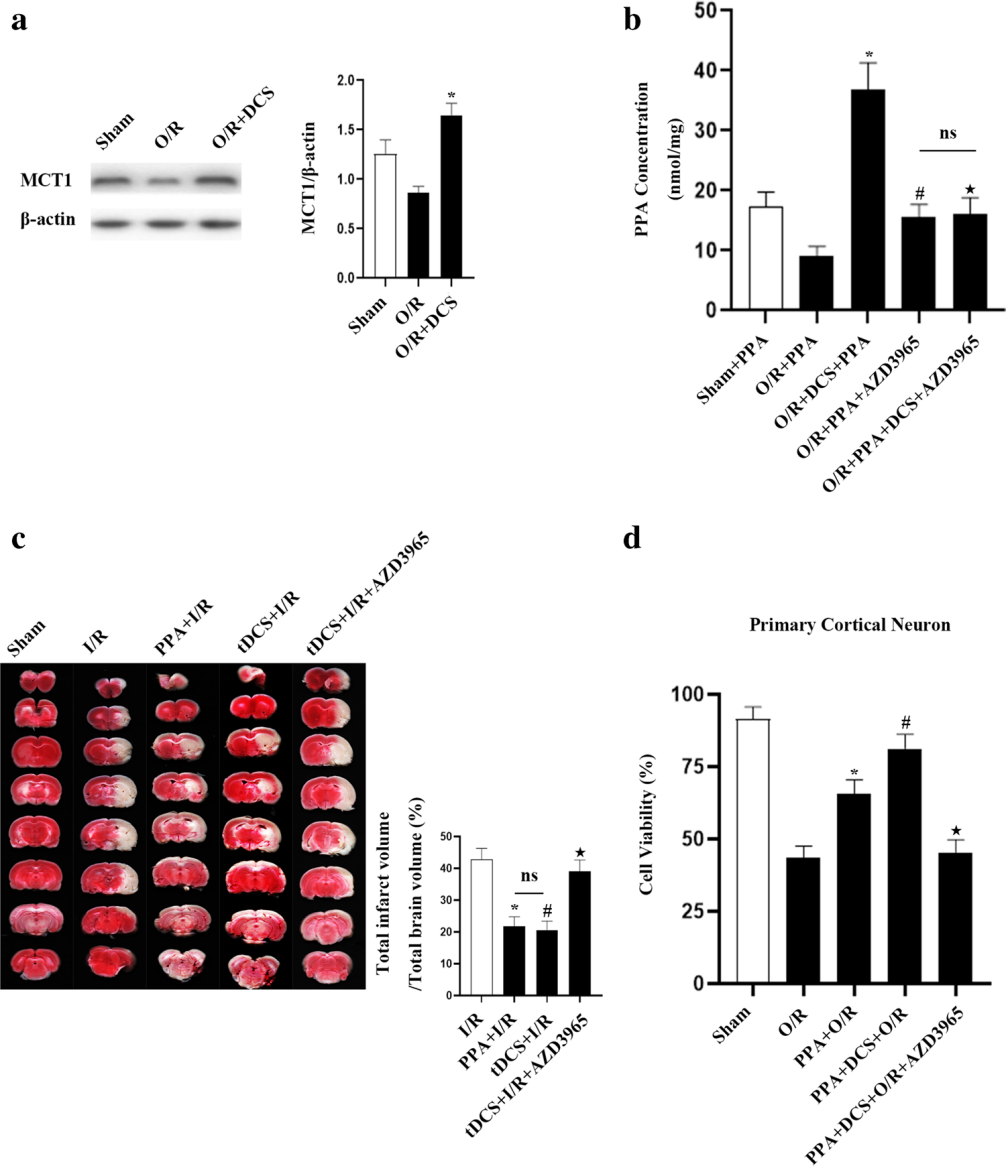
tDCS decreases ischemia neuronal death by increased MCT1 expression and the levels of intraneuronal PPA. **a** Western blotting revealed decreased MCT1 expression within neurons following OGD injury, while this expression was increased following DCS treatment. DCS was performed as detailed above, and at 6 h post-reoxygenation, primary cultured cortical neurons were isolated for analysis (*n* = 6/group, F (2, 15) = 11.88, ^*^p < 0.05 vs. O/R, one-way ANOVA). **b** HPLC analyses of intraneuronal PPA in primary cultured neurons revealed reductions in these levels following MCT1 inhibition and increased in these levels following DCS treatment. Primary neurons were treated with AZD3965 at the initiation of hypoxic brain injury (*n* = 6/group, F (4, 25) = 14.04, ^*^p < 0.05 vs. OGD + PPA, ^#^*p* < 0.05 vs. DCS + OGD + PPA. ^★^*p* < 0.05 vs. OGD + DCS + PPA, one-way ANOVA). **c** tDCS treatment increased MCT1 and thereby reduces infarct size following ischemic stroke. All tDCS treatment was performed in rats at 3 h post-reperfusion, with AZD3965 being intraperitoneally injected prior to surgery and PPA (10 mg/kg) additionally being intraperitoneally injected 1 h post-reperfusion. Brain tissue samples were selected for TTC staining at 23 h post-reperfusion (*n* = 6/group, ^*^p < 0.05 vs. I/R, ^#^*p* < 0.05 vs. I/R, ^★^*p* < 0.05 vs. tDCS + I/R, one-way ANOVA). **d** Cell viability assays indicated that DCS treatment increased MCT1 and thereby reduces neuronal death. DCS treatment was performed as above, and AZD3965 treatment of primary neurons was performed at the start of OGD. Following a 1-h reoxygenation period, neurons were treated with PPA (*n* = 6/group, F (4, 25) = 22.61, ^*^p < 0.05 vs. O/R, ^#^*p* < 0.05 vs. PPA + O/R, ^★^*p* < 0.05 vs. PPA + DCS + O/R, one-way ANOVA)

We first found that DCS was able to increase MCT1 levels on neuronal membranes, in addition to increasing levels of PPA within cultured neurons (Figs. 7a, b). MCT1 inhibitor treatment decreased intraneuronal PPA concentrations; however, administering DCS did not further increase intraneuronal PPA levels (Fig. 7b). This indicated that DCS may increase the levels of PPA within neurons by increasing MCT1 expression on ischemic neuronal membranes.

## tDCS Protects against Ischemic Neuronal Death through Increase of PPA Level

As cathodal tDCS has been shown to protect against cerebral ischemic injury [18], we next sought to determine whether such tDCS-mediated neuroprotective functions occur in an MCT1-dependent manner. To that end, we initially confirmed that cathodic tDCS exhibited neuroprotective effects in our rat MCAO model system (Fig. 7c), and we then demonstrated that exogenous PPA administration increased these neuroprotective effects for both DCS and tDCS in our OGD and MCAO model systems. We did, on the other hand, find that MCT1 inhibitor, AZD3965, eliminated the neuroprotective effects after ischemic intensity or associated neuronal injury following DCS or tDCS application (Fig. 7c–d, Supplementary Fig. 4). Together, these results suggest that tDCS can increase the level of MCT1, thereby reducing the degree of associated ischemic neuronal injury.

## Discussion

Undigested dietary ingredients such as dietary fiber and starch are transported to the lower digestive tract where they are fermented by anaerobic bacteria to produce short-chain fatty acids, such as acetic acid, propionic acid, and butyric acid [1]. Moreover, SCFAs will be almost completely absorbed by the intestine, thus providing energy to the body. In addition, SCFAs can also affect gastrointestinal function, colonic blood flow [25], pancreatic secretion [26], intestinal mucosal growth [27], and cholesterol levels [28]. With that said, the precise role of PPA in the context of ischemic stroke remains unclear. Our present analyses highlighted a non-canonical role for PPA in a rat model of cerebral I/R injury, revealing decreased levels of this PPA within the CSF following MCAO modeling. Consistently, the concentration of intraneuronal PPA decreased following the OGD treatment of cultured primary neurons. Increasing these intraneuronal PPA levels suppressed OGD-induced neuronal death, whereas the lack of PPA exacerbated such injury. As such, the intraneuronal accumulation of PPA represents a viable therapeutic target for the treatment of ischemic stroke patients.

MCT1 is a monocarboxylic acid transporter in the cerebral microvascular endothelium. Factors that regulate MCT1 function may be critical in controlling the extent of lactic acidosis and, consequently, brain damage, during stroke [29]. Previous studies have shown that electroacupuncture upregulates astrocytic MCT1 expression to improve neurological deficit in middle cerebral artery occlusion rats [30]. However, the regulatory mechanisms of MCT1 in cerebral ischemia remain undefined. Herein, we found that PPA was able to aggravate neuronal death through a mechanism dependent on decreased MCT1 expression on neurons following OGD injury.

PTEN phosphatase was first identified as a potent tumor suppressor [31], but was more recently demonstrated by Wan et al. to prevent neuronal death when downregulated by preserving GABA(A)R function in ischemic stroke [32]. In this study, we found that inhibiting the expression of PTEN in neurons reduced the death of neurons after I/R injury. In addition, we proved that the increase of PPA reduced the level of PTEN in neurons and that PTEN was connected with the process of PPA-induced death of neurons after OGD.

LONP1 is one of the major proteases patrolling the mitochondrial matrix and is also a mitochondrial protease and chaperone located in the mitochondrial matrix. Initial studies classified LonP1 as a “stress response protein,” which are upregulated in response to cellular stress [33]. More recently, LONP1 has been involved in the control of mitochondrial metabolic networks in melanoma cells as well as being involved in hypoxia adaptation in glioma cells. Researchers have demonstrated that AKT phosphorylation enhances the protease activity of mitochondrial LONP1 under hypoxia conditions [34]. The role of LONP1 in the context of ischemic stroke, however, remains to be elucidated. Herein, we provided novel evidence that reduction of LONP1 expression within ischemic neurons results in neuronal death following I/R injury. Moreover, we found that increases in PPA levels result in increases in LONP1 levels within ischemic neurons. This mediates OGD-induced neuronal death in response to PPA. In this study, we found that LONP1 expression increased after PTEN expression was inhibited, whereas LONP1 inhibited failed to impact PTEN expression. PTEN inhibited induced neuroprotective effects of LONP1 against I/R-induced injury. These results thus suggest that PTEN can regulate neuroprotective activities via multiple mechanisms including the PTEN-LONP1 signaling axis. With that said, further research still is requited to fully clarify the underlying molecular mechanisms.

The tDCS technique relies on the stimulation of cortical regions by emitting a low-intensity (1–2 mA) direct-current that can enhance or suppress neurological activity in a noninvasive, pain-free, easy-to-implement approach. Clinical studies have demonstrated that tDCS can be effectively used to treat conditions including chronic pain, schizophrenia, stroke, depression, and Parkinson’s disease [17]. Herein, tDCS was found to exert neuroprotective efficacy in the context of cerebral I/R injury by regulating the PPA-dependent signaling pathway. These results highlight the promise of tDCS as a neuroprotective treatment option for stroke patients.

In summary, this study for the first time demonstrates that cerebral ischemia injury results in decreased levels of intraneuronal PPA, which promotes neuronal cell death. We provided novel evidence that the tDCS acts on the MCT1-PPA-PTEN-LONP1 signal pathway to protect against neuronal death following cerebral I/R injury. These results support PPA could become an attractive candidate for stroke treatment and revealed a potential application of tDCS for the treatment of ischemic stroke.

## Materials and Methods

### Animals

All animal experiments were performed in accordance to the IACUC guidelines of Qingdao University School of Medicine. All animals used and experimental protocols were approved and carried out in accordance with the IACUC guidelines and the Qingdao University Animal Protection and Ethics Committee. All animal studies were conducted in accordance with the Guidelines for Reporting Animal Experiments [35]. Male adult Sprague–Dawley (SD) rats, 250 g (Qingdao Daren Fucheng Animal Husbandry Co., Ltd.), were housed in 2–3 rats per cage with a 12-h light/dark cycle, at 23–25 °C in a temperature-controlled room with free access to food and water. The animals were given more than 3 days to acclimatize before the experiment. After ischemia–reperfusion, using a 1-cm syringe, cerebrospinal fluid (≈50 μL) was collected from the foramen magnum of the occipital bone at 0 h and 6 h in rats fixed with a 27-mm winged needle [36]. Samples were randomly assigned to experimental groups using a randomization method, and data collection and processing were performed. Animal handling and all experiments were performed in accordance with international guidelines for animal welfare, and measures were taken to minimize animal pain and discomfort. The grouping of animals was conducted as a blinded experiment, and the researchers did not know the grouping information.

## Experimental Groups

Experiment 1: To detect PPA in cerebrospinal fluid and neuron, we divided the rats into Control, Sham, and I/R and the neuron into Control + PPA, Sham + PPA, O/R + PPA, and O/R + PPA + AZD3965. In order to further detect the content of PPA in neuron after DCS treatment, we divided the neuron into Sham + PPA, O/R + PPA, O/R + PPA + DCS, and O/R + PPA + DCS + AZD3965 (*n* = 6 per group).

Experiment 2: To study the effect of PPA on ischemic injury, we divided the rats into Sham, I/R, I/R + PPA, and I/R + AZD3965 and the neuron into Sham, O/R, O/R + PPA, and O/R + PPA + AZD3965. To study the protective effect of tDCS, we divided the rats into Sham, I/R, tDCS + I/R, and tDCS + I/R + AZD3965 and the neuron into Sham, O/R, O/R + PPA, O/R + PPA + DCS, and O/R + PPA + DCS + AZD3965.

Experiment 3: To investigate the signaling pathways of PPA for protection against ischemic injury and to detect the neuroprotective effects of LONP1 and PTEN, we divided the rats into Control, Sham, I/R 3 h, and I/R 6 h and the neuron into Control, Sham, O/R 3 h, and O/R 6 h, O/R + PPA, O/R + PPA + bortezomib, O/R + PPA + BpV(pic).

Experiment 4: To verify that PPA functions through the PTEN/LONP1 signaling pathway, we divided the neuron into Sham, O/R, O/R + siRNApten, and O/R + PPA.

Experiment 5: To demonstrate that tDCS exerts neuroprotective effects by increasing PPA content, we divided the neuron into Sham, O/R, and O/R + DCS.

## Transient Focal Cerebral Ischemia

A rat model of transient focal cerebral ischemia was operated on using the suture occlusion technique described in our previous study [35, 37]. Male SD rats weighing 250–300 g were anesthetized under a gas mixture of 4% isoflurane, 70% N_2_, and 30% O_2_. Their body temperatures were maintained at a constant degree intra- and postoperatively using an even heat blanket. An incision was first made through the middle of the neck, and the right external carotid artery (ECA) was resected. A 6–0 nylon suture was carefully inserted through the ECA into the right internal carotid artery to block the beginning of the right middle cerebral artery (MCA). After 1 h of blockage, the suture was removed for reperfusion, the ECA was ligated, and the wound was finally closed. The sham-operated group of rats underwent the same procedure, the difference being that the sutures were not inserted into the internal carotid artery [21, 38]. The body temperature was maintained at 37.0 ± 0.5 °C using an even heat blanket and a heat lamp. Twenty-four h after middle cerebral artery occlusion (MCAO), brain tissue specimens were obtained, anaesthetized, and executed at various times depending on the need for reperfusion. Peri-infarct tissue samples, from the ipsilateral cerebral hemisphere, was obtained for Western blotting. Subsequently samples were frozen and stained with TTC (2,3,5-triphenyltetrazolium). In appropriate experiments, an intravenous injection of AZD3965 (10 μM, 1 μL, S80362, Yuanye, Shanghai) was delivered prior to MCAO modeling, while propionic acid (0.2 mL/10 g, 200 mM, Sigma-Aldrich) was intraperitoneally injected 1 h after MCAO injury.

## TTC Staining and Infarct Volume Measurement

2, 3, 5-triphenyltetrazoliumchloride (TTC, Sigma, USA) was used to determine and infarct volume and was performed as previously described [38]. Animals were executed 24 h after surgical reperfusion in MCAO. After which, rats were perfused with 0.9% saline, and brain tissue was quickly removed in a cooled matrix. Two-mm coronal slices were obtained to calculate infarct volume (in mm^3^). Brain slices were stained with 2% TTC in phosphate-buffered saline at 37 °C for 30 min and fixed overnight in 4% paraformaldehyde (PFA) at 4 °C. Normal brain areas appeared red in color, while infarcted areas were not stained. After filming with a scanner, all data were collected together, and the areas of the cerebral infarct, ipsilateral cerebral hemisphere, and contralateral cerebral hemisphere were measured using an image analysis software (NIH Image J). The edema-corrected cerebral infarct area (area of the contralateral cerebral hemisphere minus the area of healthy tissue in the ipsilateral cerebral hemisphere) was integrated to yield the infarct volume.

## Primary Cortical Neuron Culture

Female SD rat embryos were subjected to cortical neuronal culture on day 17, as we previously described [39]. Briefly, the embryonic brain was isolated, the meninges were stripped and digested with 0.05% trypsin at 37 °C for 20 min, and the isolated neurons were suspended in a plate medium (neural basal medium, 2% B-27 additive, 0.5% fetal bovine serum, 0.5 μM L-glutamine, and 25 μM glutamic acid). Subsequently, the obtained tissue was inoculated in Petri dishes coated with poly-D-lysine. After 1.0 day of incubation, half of the medium was replaced with the maintenance solution (neural basal medium, 2% B-27 supplement, 0.5 mM L-glutamine). Thereafter, the maintenance solution was changed every 3.0 days in the same way. Cultured neurons were used for experiments 12 days after inoculation. The grouping of neuron was done using a blinded experiment where the researchers did not know the grouping information.

## Oxygen–Glucose Deprivation Insult

The hypoxia-glucose deprivation insult (OGD) method was described according to our previous study [40]. Prior to initiating OGD, cells were transferred to glucose-free deoxygenated extracellular solution (ECS) (in mM: 116 NaCl, 5.4 KCl, 0.8 MgSO_4_, 1.0NaH_2_PO_4_, 1.8 CaCl_2_, and 26 NaHCO_3_), placed in a dedicated incubator, and maintained at 37 °C, 95% N_2_, and 5% CO_2_ for 2 h. Neurons were removed from the incubator, transferred to the maintenance culture, and returned to the incubator. For the Sham group, cultures were transferred to standard ECS (in mM: 116NaCl, 5.4KCl, 0.8MgSO_4_, 1.0NaH_2_PO_4_, 1.8CaCl_2_, 26NaHCO_3_, and 33 glucose) and introduced into a chamber maintained at 37 °C, 95% air, and 5% CO_2_. After 2 h of incubation, the neurons were transferred to the maintenance culture solution and returned to the original incubator. Propionic acid (1 mM), AZD3965 (10 μM, 1 μL, S80362, Yuanye, Shanghai), and bortezomib (4 nM, Sigma-Aldrich) were given 1 h after reoxygenation of glucose deprivation. Bisperoxovanadium (pyridine-2-carboxyl) [BpV(pic)] (Santa Cruz, CA) was applied to primary neurons 30 min before OGD injury.

## Western Blotting Analysis

Western blotting was performed according to our previously described method [41]. Total proteins were extracted with lysate, and equal amounts of proteins were separated by 10% SDS polyacrylamide gel electrophoresis (SDS-PAGE). The proteins were transferred to polyvinylidene difluoride membranes, and the membranes were closed with 5% (w/v) bovine serum albumin or 5% (w/v) skimmed dry milk in TBST (containing 0.1% TBS Tween 20). They were incubated for 1 h at room temperature, followed by overnight incubation at 4 °C with primary antibody and 1 h at room temperature with HRP-coupled. The antigen–antibody complexes were detected by chemiluminescence reagents (microtiter wells). Primary antibodies LONP1 (rabbit, 1:1000, A4293, ABclonal, China), MCT1 (rabbit, 1:1000, A3013, ABclonal, China), PTEN (rabbit, 1:100, 9188, Cell Signaling Technology, USA), and β-actin (rabbit, 1:2000, bs-0061R, Bioinformatics, China).

## Immunofluorescent Staining and Confocal Microscopy

Immunofluorescence staining was performed using our previous method [42]. Frozen brain sections, 30 μm thick, were fixed in 4% paraformaldehyde (PFA), rinsed three times with PBS, subsequently closed for 2 h in 10% goat serum, and incubated in specific primary antibodies overnight at 4 °C. The primary antibodies used were LONP1 (rabbit, 1:500, A4293, ABclonal, China) and MAP2 (mouse, 1:500, Sigma, M4403). After rinsing with PBS for 3 h, the sections were incubated with Goat anti-mouse IgG-AlexaFluor-488 conjugate and Goat-anti-rabbit IgG-AlexaFluor-555 secondary antibodies for 2 h. The sections were washed 3 times with PBS, sealed and dried in an oven at 30–32 °C for 10–15 min, and used for brain section observation using a fluorescent confocal microscope (C2Si, Nikon, Tokyo, Japan).

Primary cultured neuronal cells were inoculated in 24-well culture plates, equipped with cell crawls, and then, immunofluorescence staining was performed. Cells were fixed in 4% paraformaldehyde (PFA) for 20 min. Fixed cells were incubated for 2 h in a blocking buffer (5% donkey serum, 0.3% Triton X-100 in PBS). We incubated the samples in specific primary antibodies (anti-LONP1, MAP2) overnight at 4 °C. The cells were incubated with Goat anti-mouse IgG-AlexaFluor-488 conjugate and Goat-anti-rabbit IgG-AlexaFluor-555 secondary antibodies for 2 h. The cells were washed 3 times with PBS. Antibodies were incubated with Goat anti-mouse IgG-AlexaFluor-488 conjugate and Goat-anti-rabbit IgG-AlexaFluor-555 secondary antibodies for 2 h. The samples were then washed 3 times with PBS. Finally, glass coverslips or glass slides were dried in an oven at 30–32 °C for 10–15 min and used for cell observation using a fluorescence confocal microscope (C2Si, Nikon, Tokyo, Japan). All images were processed using the same settings.

## Subcellular Fractionation Assays

A Membrane, Nuclear, and Cytoplasmic Protein Extraction Kit (Solarbio Inc., Beijing, China) was used for subcellular fractionation [42]. Briefly, neurons were collected, rinsed with 500 uL of cold PBS, and combined with 1 mL of ice-cold solution A (containing 1-μL protease inhibitor, 5-μL phosphatase inhibitor, 1-μL DTT, and 10-μL PMSF, freshly added). A glass homogenizer (30–50 cycles) or a sonicator (30 s, 1 min interval) was used to homogenize samples, with this process being repeated three times. The absence of cell clumps was used to confirm homogenization efficiency, after which the mixture was vortexed for 10 s and incubated for 20 min on ice, shaking 3–5 times during this period. Samples were then centrifuged for 10 min at 12,000 rpm, and supernatants containing the cytoplasmic fraction were transferred for fresh tubes at stored at − 80 °C. The remaining precipitates were then suspended in 500 uL of ice-cold solution B, mixed vigorously for 10 s, and incubated on ice for 20 min, as well as being shaken 3–5 times during the period. Samples were then centrifuged for 10 min at 12,000 rpm, after which 500 uL of ice-cold solution C was added to the precipitates; then the above incubation and centrifugation steps were again repeated. Supernatants containing the membrane fraction were then transferred to fresh tubes and stored at − 80 °C. Western blotting was used to detect MCT1 within the membrane fraction, while GAPDH served as an internal control for the cytoplasmic fraction.

## High-Performance Liquid Chromatography (HPLC)

The cerebrospinal fluid from the foramina magnum was homogenized with 200-μL cold normal saline (0.9%) and centrifuged at 140,000 RPM at 4 °C for 20 min. The supernatant was derived by adding 1-mL 0.15 mM H_2_SO_4_ solution. Separation was conducted at 35 °C. The HPLC system was operated in gradient mode by binary gradient elution of (A) 10 mM H_2_SO_4_ and (B) phosphoric acid buffer at a flow rate of 0.4 mL/min. Gradient scheme: 0 min A: 5%, 6 min A: 20%, 12 min A: 30%, 16 min A: 30%, 25 min A: 40%, 28 min A: 80%, 32 min A: 80%, 35 min A: 5%. The chamber temperature was 30 °C, and the detection wavelength was 263 nm. The injection volume was 10 μL. Under specified experimental conditions, a single PPA was detected according to the established PPA retention time. We calculated the subpeak area of a given bile acid from the known concentration.

## Lentivirus Transfection

The PTEN siRNA (siRNApten) and non-targeting control siRNA (NsiRNA) was purchased from Santa Cruz Biotechnology, Santa Cruz, CA, USA. The sequence of human PTEN siRNA was 5′-CTG CTA GCC TCT GGA TTT GA-3′, and non-targeting control siRNA was 5′-CTT CTG GCA TCC GGT TTA GA-3′.

## DCS and tDCS Application

In the OGD model, DCSs were applied to neurons cultured in culture chambers using the method we described earlier [43]. For DCS stimulation, agar-salt bridges were used to connect silver/silver chloride electrodes in beakers to pools of culture medium on either side of the chambers. Control culture conditions were identical except that DCSs were not added. HEPES acid (20 mM) was added to the medium, and the pH was adjusted to 7.4. Cells were stimulated with DCS at a current strength of 250 mV/mm for 20 min at 3 h after OGD reoxygenation [19].

In the middle cerebral artery obstruction (MCAO) model, rats were applied tDCS while awake with a constant current stimulator (Schneider Electronics, Gleichen, Germany), which was made specifically for animals to apply low-intensity currents [44]. tDCS was applied to the rat MCAO model using the method we described previously [19]. The MCAO procedure was performed 7 days after performing skull base electrode implantation. One electrode was fixed symmetrically on both sides of the skull with a nontoxic glass ionomer. The electrode at the ischemic cortex was connected to the cathodic end, and the other electrode was connected to the anodic end. The electrodes were filled with saline prior to stimulation. Rats were treated with tDCS at a current density of 2.86 mA/cm^2^, while the current intensity was 100 μA. At 3 h after I/R, rats were stimulated for 10 min, rested for 3 min, and then stimulated again for 10 min, for a total of 8 times [45]. The rats in the sham-operated group underwent the same procedure as the stimulated group, except that no current was applied.

## Analysis of Lactate Dehydrogenase Release and Cell Viability

Neuronal survival, or injury, was assessed using a Cell Counting Kit 8 (CCK8; Dojindo, CK04) or the Lactate Dehydrogenase Release Assay Kit (Biovision, K311-400). Both methods are mentioned the manufacturer’s instructions. CCK-8 assay: Add 10 μL of CCK8 reagent, incubate for 2 h, and measure the absorbance value at 450 nm using a 96-well plate reader (Molecular Devices, USA). LDH assay: Measures LDH levels by analyzing the amount of LDH released from cells into the culture medium. Using a 96-well plate reader, absorbance values at 490 nm were measured, and cell viability was calculated according to the manufacturer’s instructions [42].

## Statistics

GraphPad Prism was used for all statistical testing. The group sizes per experiment was based on a power analysis [46]. Data were compared via Student’s *t*-tests or ANOVAs and are given as means ± SEM. *p* < 0.05 was the significance threshold.

## Supplementary Information

Below is the link to the electronic supplementary material.

Supplementary file1 (DOCX 1433 KB)

Supplementary file2 (DOCX 17 KB)

## Data Availability

The data that support the findings of this study are available from the corresponding author upon reasonable request. Some data may not be made available because of privacy or ethical restrictions.
