# Supplementary material for: Transcranial Direct-Current Stimulation Regulates MCT1-PPA-PTEN-LONP1 Signaling to Confer Neuroprotection After Rat Cerebral Ischemia–Reperfusion Injury
Source: Mol Neurobiol. 2022 Oct 3;59(12):7423–38. doi: 10.1007/s12035-022-03051-7 (PMC9616768; doi:10.1007/s12035-022-03051-7)
Supplement: Supplementary file 1 — Supplementary file1 (DOCX 1433 KB) [file 12035_2022_3051_MOESM1_ESM.docx]

**Transcranial direct-current stimulation regulates MCT1/PPA/LONP1/PTEN signaling to confer neuroprotection following rat cerebral ischemia-reperfusion injury**

Xiangyi Kong^a^, Wenjie Hu^a,b^, Yu Cui^a^, Jingchen Gao^a^, Xujin Yao^a^, Jinyang Ren^a^, Tao Lin^a^, Jiangdong Sun^a^, Yunyi Gao^a^, Xiaohua Li^a^, Hui Wang^a^, Huanting Li^a^, Fengyuan Che^c^, Qi Wan^a,d^

*^a^Institute of Neuroregeneration & Neurorehabilitation, Department of Neurosurgery, Qingdao University, 308 Ningxia Street, Qingdao 266071, China*

*^b^Department of Biological Science, Jining Medical University, Rizhao, Shandong, China.*

*^c^Central Laboratory, Department of Neurology, Linyi People's Hospital, Qingdao University, 27 East Jiefang Road, Linyi, Shandong, China*

*^d^Qingdao Gui-Hong Intelligent Medical Technology Co. Ltd, 7 Fenglong Road, Qingdao High-tech Industrial Development District, Qingdao, China*

Correspondence:

Qi Wan, Institute of Neuroregeneration & Neurorehabilitation, Department of Neurosurgery, Qingdao University, 308 Ningxia Street, Qingdao 266071, China. Email: qiwan1@hotmail.com

Fengyuan Che, Central Laboratory, Department of Neurology, Linyi People's Hospital, Qingdao University, 27 East Jiefang Road, Linyi, Shandong, China

Email: che1971@126.com

**Running title:** Regulation of MCT1-PPA-PTEN-LONP1 signaling by tDCS

**Keywords:** Propionic acid; cerebral ischemic stroke; neuroprotection; LONP1; tDCS


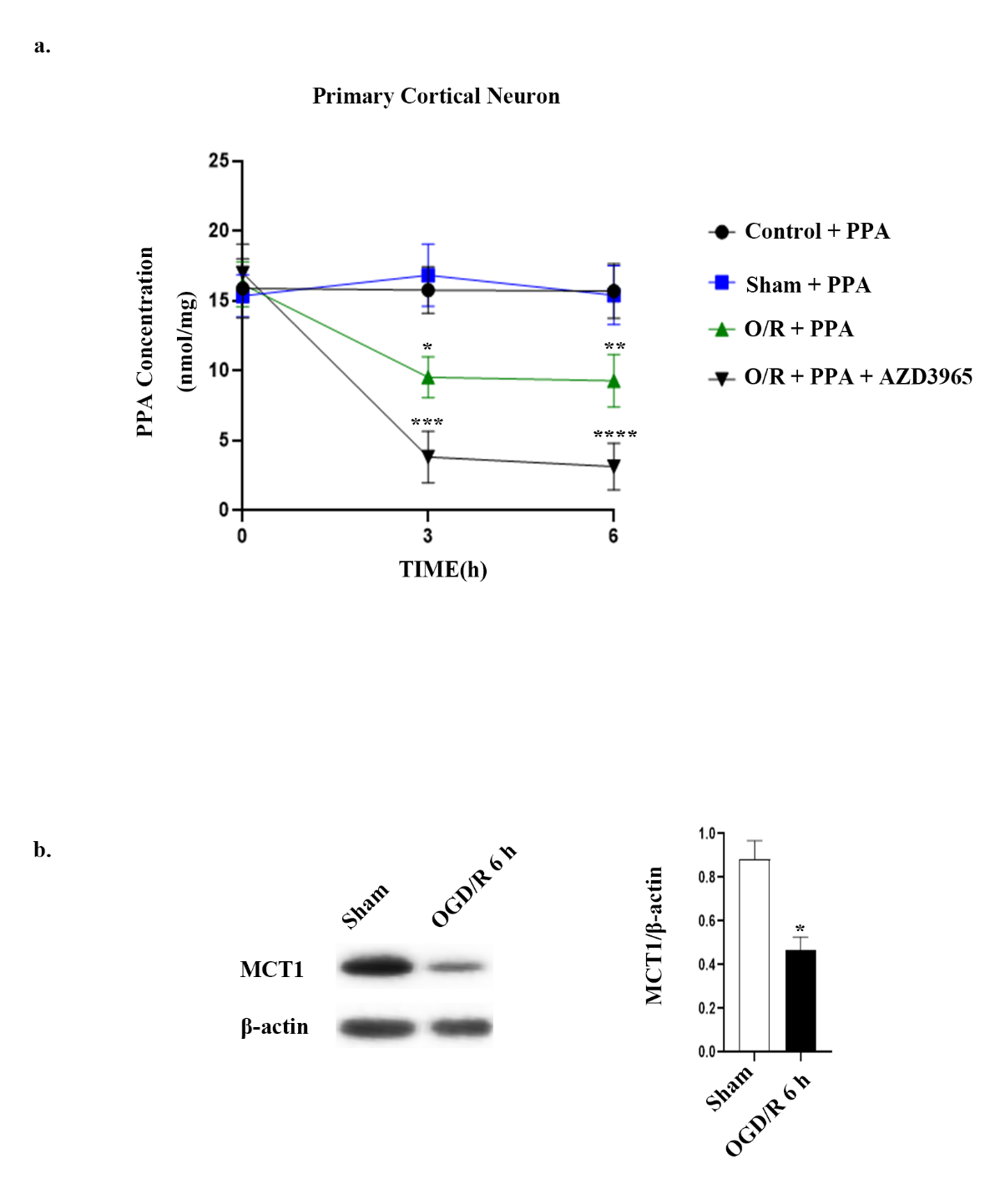


**Supplementary Fig. 1. OGD-induced decreases in membrane MCT1 levels result in decreased intraneuronal PPA concentrations**

1. HPLC analyses were used to measure PPA levels in primary neuron cultures at 0, 3, and 6 h following OGD treatment and reperfusion, revealing decreases in intraneuronal PPA levels following I/R injury. Cells in the OGD+PPA+AZD3965 group were treated with AZD3965 at the initiation of the replacement of extracellular solution (n=6/group, ^*^p < 0.05 vs. 3 h sham, ^**^p< 0.05 vs. 6 h sham, ^***^p< 0.05 vs. 3 h sham, ^****^p< 0.05 vs. 6 h sham, one-way ANOVA).
2. Western blotting revealed decreases in intraneuronal MCT1 levels following OGD injury. Western blotting was used to analyze primary cultured cortical neurons at 6h following re-oxygenation (n=6/group, ^*^p< 0.05 vs. sham, Student’s t-test).


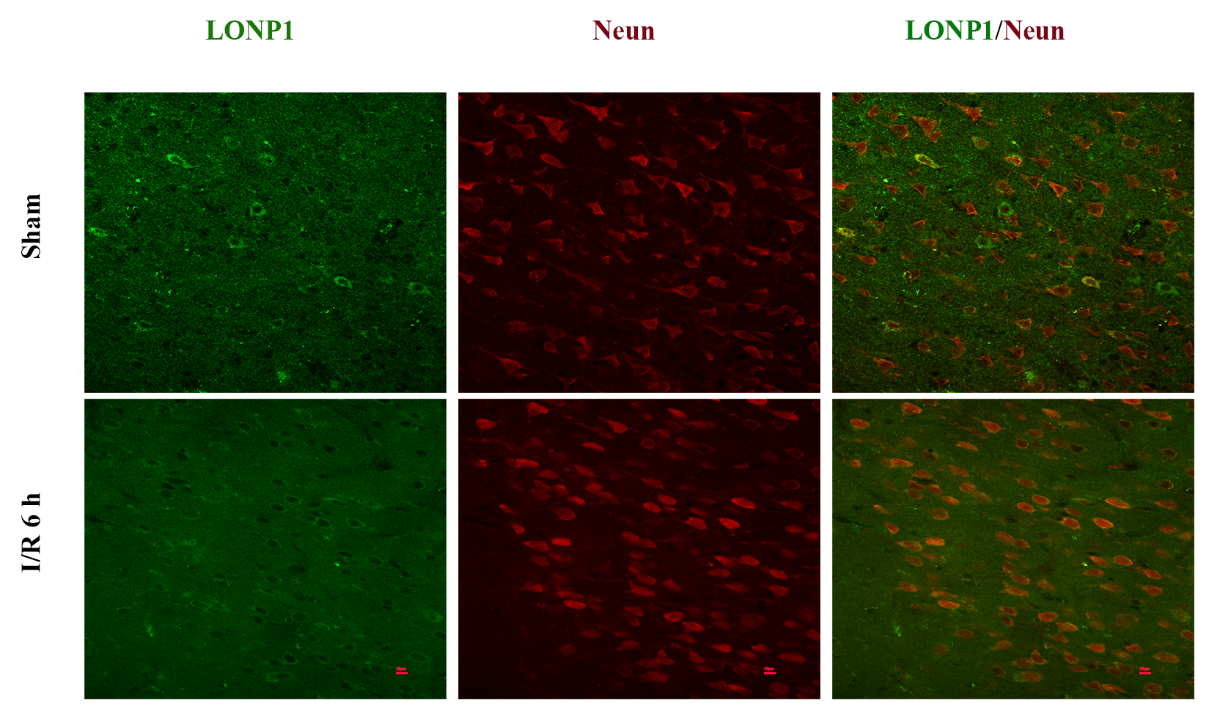


**Supplementary Fig. 2. Intraneuronal LONP1 levels are reduced following ischemic injury**

1. Immunofluorescent LONP1 staining in the cerebral cortex. Neuron (NeuN, red), LONP1 (green), (n=6/group).


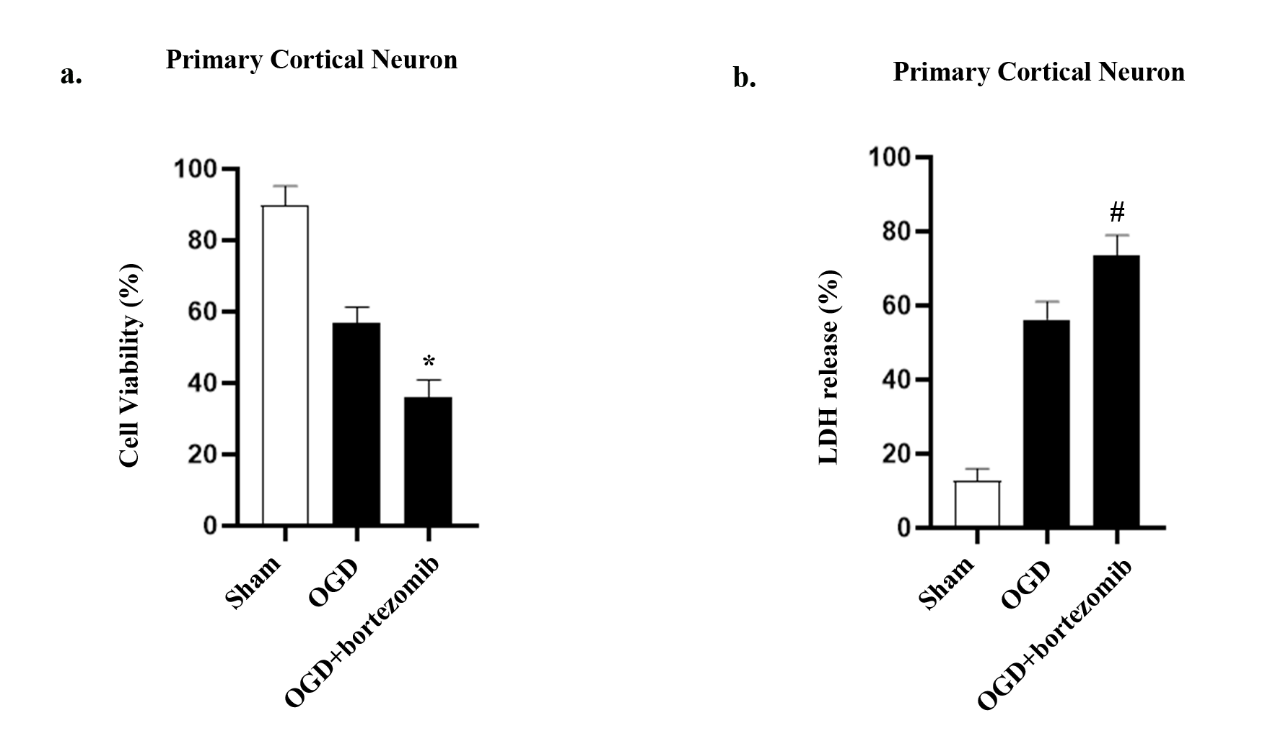


**Supplementary Fig. 3. LONP1 downregulation promoted neuronal death following OGD treatment**

1. In cell viability assays, LONP1 inhibitor bortezomib increased neuronal death following OGD treatment. Neurons were transfected with bortezomib 1 h prior to OGD treatment. (n=6/group, ^*^p < 0.05 vs. OGD, one-way ANOVA).
2. LDH release assays revealed that the LONP1 inhibitor bortezomib increases OGD-induced neuronal death. Neurons were transfected with bortezomib 1 h prior to OGD treatment. (n=6/group, ^#^p < 0.05 vs. OGD, one-way ANOVA).

**
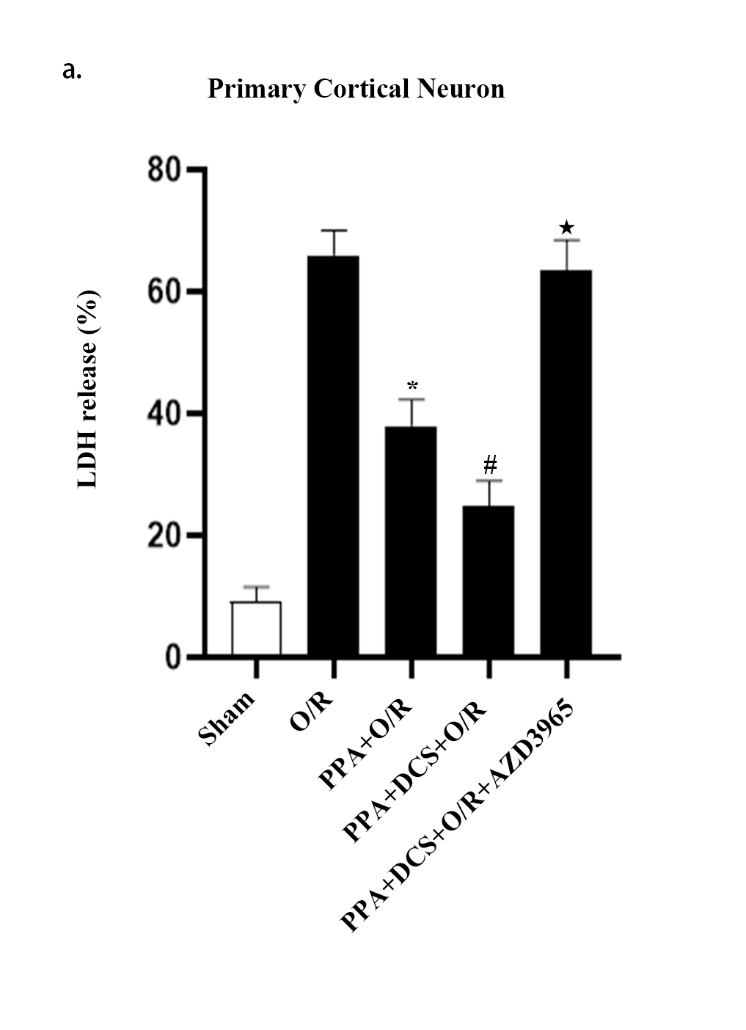
**

**Supplementary Fig. 4. tDCS prevents ischemic neuronal death by increasing MCT1 and intraneuronal PPA levels**

In LDH release assays, DCS was found to increased MCT1 and to thereby prevent neuronal death. DCS was implemented as in prior experiments. Primary neurons were treated with AZD3965 was at the initiation of OGD. Following a 1 h re-oxygenation stem, PPA (1 mM) was applied to primary neuronal cultures (n=6/group, F (4, 25) = 36.83, ^*^p < 0.05 vs. O/R, ^#^p < 0.05 vs. PPA+O/R, ^★^p < 0.05 vs. DCS + PPA + O/R, one-way ANOVA).
