# Supplementary material for: Transcranial Direct-Current Stimulation Regulates MCT1-PPA-PTEN-LONP1 Signaling to Confer Neuroprotection After Rat Cerebral Ischemia–Reperfusion Injury
Source: Mol Neurobiol. 2022 Oct 3;59(12):7423–38. doi: 10.1007/s12035-022-03051-7 (PMC9616768; doi:10.1007/s12035-022-03051-7)
Supplement: Supplementary file 2 — Supplementary file2 (DOCX 17 KB) [file 12035_2022_3051_MOESM2_ESM.docx]

**Supplementary Table**

Table1. Experimental protocols

| Grouping | Aims | In cultured neurons | In rats |
| --- | --- | --- | --- |
| Experiment 1 | Detection of PPA in cerebrospinal fluid and neuron  Detect the content of PPA in neuron after DCS treatment | Control + PPA, Sham + PPA, O/R + PPA, O/R + PPA + AZD3965  Sham + PPA, O/R + PPA, O/R + PPA + DCS, O/R + PPA + DCS + AZD3965 | Control, Sham, I/R |
| Experiment 2 | Effect of PPA on ischemic injury  Protective effect of tDCS | Sham, O/R, O/R + PPA, O/R + PPA + AZD3965  Sham, O/R, O/R + PPA, O/R + PPA + DCS, O/R + PPA + DCS + AZD3965 | Sham, I/R, I/R + PPA, I/R + AZD3965  Sham, I/R, tDCS + I/R, tDCS + I/R + AZD3965 |
| Experiment 3 | Detection of neuroprotective effects of LONP1 and PTEN | Control, Sham, O/R 3 h, O/R 6 h. O/R + PPA, O/R + PPA + bortezomib, O/R + PPA + BpV(pic) | Control, Sham, I/R 3 h,  I/R 6 h |
| Experiment 4 | PPA functions through the PTEN/LONP1 signaling pathway | Sham, O/R, O/R + siRNApten, O/R + PPA |  |
| Experiment 5 | tDCS exerts neuroprotective effects by increasing PPA concentration | Sham, O/R, O/R + DCS |  |
